# Copper-Binding Domain Variation in a Novel Murine Lysyl Oxidase Model Produces Structurally Inferior Aortic Elastic Fibers Whose Failure Is Modified by Age, Sex, and Blood Pressure

**DOI:** 10.3390/ijms23126749

**Published:** 2022-06-17

**Authors:** Kit Man Tsang, Russell H. Knutsen, Charles J. Billington, Eric Lindberg, Heiko Steenbock, Yi-Ping Fu, Amanda Wardlaw-Pickett, Delong Liu, Daniela Malide, Zu-Xi Yu, Christopher K. E. Bleck, Jürgen Brinckmann, Beth A. Kozel

**Affiliations:** 1 National Heart, Lung, and Blood Institute, National Institutes of Health, Bethesda, MD 20892, USA; kitman.tsang@nih.gov (K.M.T.); russell.knutsen@nih.gov (R.H.K.); billi020@umn.edu (C.J.B.J.); lindbergej@nih.gov (E.L.); fuy2@nih.gov (Y.-P.F.); mandywardlaw@gmail.com (A.W.-P.); liud2@mail.nih.gov (D.L.); dmalide@nih.gov (D.M.); yuz@nih.gov (Z.-X.Y.); bleckck@nih.gov (C.K.E.B.); 2 Department of Pediatrics, University of Minnesota, Minneapolis, MN 55455, USA; 3 Institute of Virology and Cell Biology, University of Lübeck, 23562 Lübeck, Germany; heiko.steenbock@uni-luebeck.de (H.S.); juergen.brinckmann@uni-luebeck.de (J.B.); 4 Johns Hopkins University Applied Physics Lab, Laurel, MD 20724, USA; 5 Department of Dermatology, University of Lübeck, 23562 Lübeck, Germany

**Keywords:** lysyl oxidase, elastin, collagen, thoracic aortic aneurysm, sex as a biological variable, Fib-SEM, aorta, two-photon, rare variant, genotype–phenotype correlation

## Abstract

Lysyl oxidase (*LOX*) is a copper-binding enzyme that cross-links elastin and collagen. The dominant *LOX* variation contributes to familial thoracic aortic aneurysm. Previously reported murine *Lox* mutants had a mild phenotype and did not dilate without drug-induced provocation. Here, we present a new, more severe mutant, *Lox^b^*^2b370.2Clo^ (c.G854T; p.Cys285Phe), whose mutation falls just N-terminal to the copper-binding domain. Unlike the other mutants, the C285F Lox protein was stably produced/secreted, and male C57Bl/6J *Lox^+/^*^C285F^ mice exhibit increased systolic blood pressure (BP; *p* < 0.05) and reduced caliber aortas (*p* < 0.01 at 100mmHg) at 3 months that independently dilate by 6 months (*p* < 0.0001). Multimodal imaging reveals markedly irregular elastic sheets in the mutant (*p* = 2.8 × 10^−8^ for breaks by histology) that become increasingly disrupted with age (*p* < 0.05) and breeding into a high BP background (*p* = 6.8 × 10^−4^). Aortic dilation was amplified in males vs. females (*p* < 0.0001 at 100mmHg) and ameliorated by castration. The transcriptome of young Lox mutants showed alteration in dexamethasone (*p* = 9.83 × 10^−30^) and TGFβ-responsive genes (*p* = 7.42 × 10^−29^), and aortas from older C57Bl/6J *Lox^+/^*^C285F^ mice showed both enhanced susceptibility to elastase (*p* < 0.01 by ANOVA) and increased deposition of aggrecan (*p* < 0.05). These findings suggest that the secreted *Lox^+/^*^C285F^ mutants produce dysfunctional elastic fibers that show increased susceptibility to proteolytic damage. Over time, the progressive weakening of the connective tissue, modified by sex and blood pressure, leads to worsening aortic disease.

## 1. Introduction

Lysyl oxidase (LOX) is an extracellular copper-dependent enzyme that catalyzes the oxidative deamination of lysine residues in collagen and elastin, resulting in spontaneous condensation with adjacent aldehydes to form inter- and intramolecule covalent crosslinks [1,2,3]. It belongs to a family of five closely related copper-dependent enzymes (LOX, LOXL1, LOXL2, LOXL3, and LOXL4). Proteins in this family have a conserved carboxy-terminal catalytic domain paired with variant amino-terminal domains. The LOX and LOXL1 propeptides must be proteolytically cleaved by procollagen C-peptidases to form an active enzyme of 30 kDa, while LOXL2–4 appear to be active in both processed and nonprocessed forms [4].

For optimal enzyme activity, three histidines in the catalytic domain must coordinate a single copper ion, causing a conformational change that enables the formation of the lysine–tyrosyl–quinone (LTQ) cofactor from lysine and tyrosine downstream from the copper-binding region [5]. In LOX, five pairs of cysteines are involved in intramolecular disulfide bonds. All the Lox cystines are evolutionarily conserved and are thought to be responsible for positioning the copper-binding domain and the LTQ near one another and stabilizing the structure [5,6].

Homozygous disruption of the *Lox* gene in mice results in death during the perinatal period due to aortic aneurysm [7,8]. These fetuses possess fragmented elastic fibers and discontinuous smooth muscle cell layers [8]. In humans, both nonsense and missense variation in *LOX*, mostly concentrated around the copper-binding domain, has been associated with FTAA in a small but growing number of pedigrees [8,9,10,11,12,13]. In general, people with *LOX*-related FTAA (using the dyadic system of naming) [14] do not exhibit vascular disease at birth but begin to develop features of aortic dilation and aneurysm over time. Dilation has been described in the reported patients as early as 6 years of age [11], but most are ascertained due to aneurysm in middle age. While most affected individuals exhibit isolated aortic aneurysm, an individual with a p.Cys291Ser variant [13] was described with multiple dissections of the aorta as well as aneurysm and dilation in extra-aortic vessels, such as the renal and iliac arteries. The orthologous amino acid (p.Cys285) was mutated to phenylalanine (Phe) as part of a mouse ENU screen identifying homozygous variants causing cardiovascular disease manifestations [15]. Our study uses this new mouse model, *Lox^b^*^2b370.2Clo^ (c.G854T; p.Cys285Phe), to investigate the progression of Lox-mediated disruption of elastic fibers over the lifetime and to evaluate potential interactive effects of sex and mechanical stress on the condition.

## 2. Results

### 2.1. Cardiovascular Properties of Young Male Lox^+/C285F^ Mice

**Figure 1:**
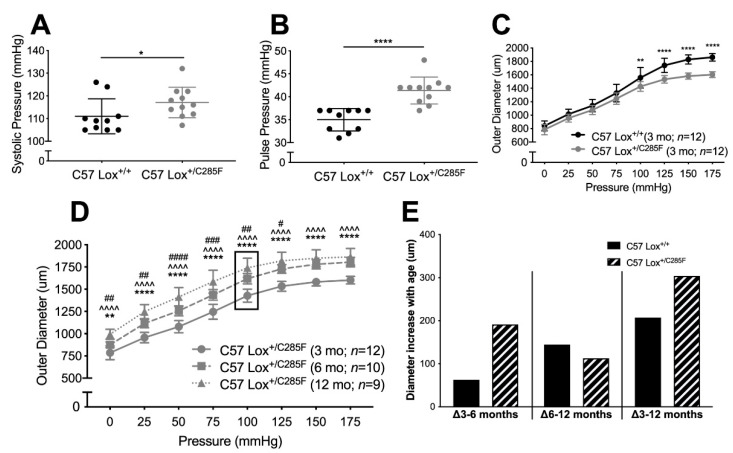
Mutation in Lox alters vascular mechanics. (**A,B**), SBP (**A**) and PP (**B**) are significantly higher in 3-month C57 Lox^+/C285F^ mice (Mann–Whitney). (**C**), Aortic diameter is significantly smaller at pressures above 100 mmHg (**C**, two-way ANOVA *p* < 0.0001 for genotype and pressure, multiple comparison testing by Sidak) but dilates with age ((**D**), *—3 vs. 6 months, ^—3 vs. 12 months, and #—6 vs. 12 months; two-way ANOVA, Sidak). (**E**), Difference of average diameters over 3–6-, 6–12-, and 3–12-month intervals show a biphasic trajectory with the average C57 Lox^+/C285F^ aorta dilating faster than C57 Lox^+/+^ early (Δ3–6 months) and leveling off later ((Δ6–12 months), for larger change in diameter over the Δ3–12-month period. For all tests *^, #^ *p* < 0.05, **^, ##^ *p* < 0.01, ^###^ *p* < 0.001, and ****^,^ ^^^^^, ####^ *p* < 0.0001.

To learn more about the pathophysiology of variation near the LOX copper-binding domain [16], we utilized a mouse with a heterozygous Lox mutation at c.G854T leading to the replacement of Cys285 (akin to Cys291 in the human sequence) with a Phe (Lox^+/C285F^). This Cys sits in close proximity to copper-binding amino acids His286 (His292), His298(His294), and His 290 (His296) (mouse (human) aa numbering) [5], and variation at this amino acid has been shown to be pathogenic in humans [13]. As expected, homozygous Lox mutation at c.G854T in mice is lethal; pups died shortly after birth (data not shown). When assessed physiologically, 3-month-old C57 Lox^+/C285F^ males show higher systolic blood pressure (SBP) than C57 Lox^+/+^ mice (average increase of 7 mmHg, Figure 1A, *p* < 0.05) with normal diastolic pressure (data not shown), leading to an average pulse pressure (PP) increase of 6 mmHg (Figure 1B, *p* < 0.0001). The implied increase in stiffness is also seen in the pressure–diameter tracings where aortas from 3-month male C57 Lox^+/C285F^ mice show decreased caliber (Figure 1C, *p* < 0.01 or better) and reduced diameter increase for each incremental pressure jump above 75 mmHg (data not shown). When allowed to age, however, C57 Lox^+/C285F^ male aortas begin to dilate (Figure 1D, note 6- and 12-month values). Some of the increases in caliber are related to ongoing growth and normal dilation with age, but when the caliber increase is calculated, the average aortic size in the Lox mutants reveals a biphasic trajectory (Figure 1E), with C57 Lox^+/C285F^ increasing in caliber more than C57 Lox^+/+^ early (Δ3–6 months) and leveling off late (Δ6–12 months). Even with that leveling off, the degree of dilation is greater in the C57 Lox^+/C285F^ mutants over the 3–12-month window.

### 2.2. Lox^+/C285F^ Dilation Rate Is Modified by Sex and Blood Pressure

**Figure 2:**
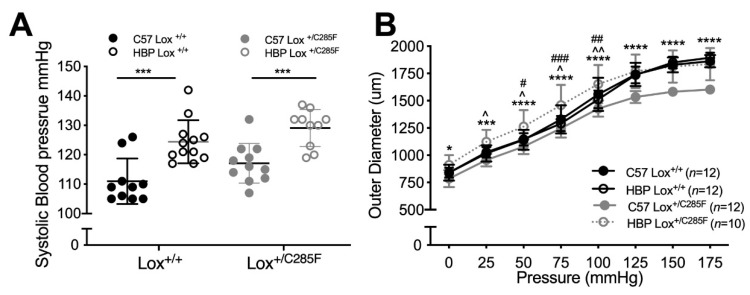
HBP congenic background increases male Lox^+/C285F^ aortic dilation rate. (**A**,**B**), HBP background increases SBP in Lox^+/+^ and Lox^+/C285F^ mice ((**A**), Lox effect *p* < 0.05, HBP effect *p* < 0.0001, two-way ANOVA, followed by Sidak multiple testing with *p* values shown in the graph) and produces larger diameter vessels in Lox^+/C285F^ males by 3 months of age ((**B**), *—C57 Lox^+/C285F^ vs. HBP Lox^+/C285F^, ^—C57 Lox^+/+^ vs. HBP Lox^+/C285F^, and ^#^—HBP Lox^+/+^ vs. HBP Lox^+/C285F^, two-way ANOVA). C57 Lox^+/+^ and C57 Lox^+/C285F^ mice are those used in Figure 1. For all tests *^,^ ^^, #^ *p* < 0.05, ^^^, ##^ *p* < 0.01, ***^, ###^ *p* < 0.001, and **** *p* < 0.0001.

Because hypertension has been shown to be a modifier of dilation rate in other models of aortic dilation [17] (and even in other Lox mutant lines [18]), we crossed the C57 Lox^+/C285F^ onto a congenic high blood pressure (HBP) background. As expected, HBP background increases SBP (Figure 2A, HBP effect *p* < 0.0001 by two-way ANOVA, see figure for multiple comparison testing (Sidak)), with an average increase of 12 mmHg at 3 months of age. Likewise, the aortic diameter in the male HBP Lox^+/C285F^ is larger at all pressures tested than the C57 Lox^+/C285F^ (Figure 2B, *p* < 0.05 or better at each pressure) and appears dilated as compared to C57 Lox^+/+^ and HBP Lox^+/+^ mice at a subset of pressures. Of note, no diameter change is seen in the HBP Lox^+/+^ aortas.

**Figure 3:**
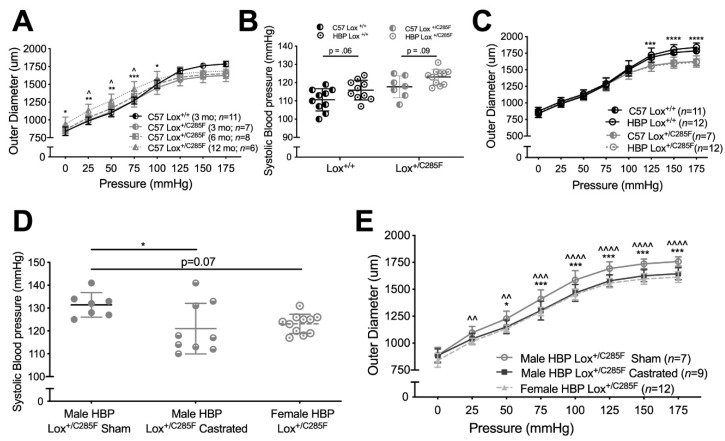
Sex effects of Lox^+/C285F^. (**A**), Female C57 Lox^+/C285F^ show no increase in diameter at 6 months relative to 3 months and only begin to show dilation by 12 months (* *p* values for 3 vs. 12-month C57 Lox^+/C285F^ and ^ *p* values for 6 vs. 12-month C57 Lox^+/C285F^ by two-way ANOVA, Tukey). (**B**,**C**), When bred to HBP, SBP was modestly increased ((**B**), *p* < 0.001 for blood pressure effect and *p* < 0.01 for Lox effect, two-way ANOVA, Sidak) but there was no HBP effect on vessel diameter (**C**, *p* = NS for HBP effect and *p* < 0.001 for Lox effect by two-way ANOVA, Tukey), * shown are for C57 Lox^+/C285F^ vs. C57 Lox^+/+^ and HBP Lox^+/C285F^ vs. HBP Lox^+/+^. There are no statistical differences in diameter within Lox genotypes. (**D**,**E**), Male HBP mice (Lox^+/C285F^ and Lox^+/+^) were castrated at P20 and assessed for SBP and aortic diameter at 3 months. SBP was lower in the castrated males ((**D**), *p* < 0.05 by one-way ANOVA, Dunn), and aortic diameter was comparable to the nondilated HBP Lox^+/C285^ female (**E**). *p* values shown are between the sham males and either the castrated males (*) or females (^). For castrated male HBP Lox^+/C285F^ vs. female HBP Lox^+/C285F^ *p* = NS. All female HBP Lox^+/C285F^ are equivalent. *^,^ ^ *p* < 0.05; **^,^ ^^ *p* < 0.01; ***^,^ ^^^ *p* < 0.001; ****^,^ ^^^^ *p* < 0.0001.

When similar testing is done in female mice, a different pattern emerges. The female C57 Lox^+/C285F^ mice do not show dilation until 12 months of age (Figure 3A). Adding the HBP background yields both HBP and Lox effects to SBP (Figure 3B, two-way ANOVA, *p* < 0.01 and *p* < 0.001, respectively, multiple comparison testing results shown in graph, Sidak). However, the HBP-mediated SBP increase is less robust in females and does not increase aortic caliber in the 3-month HBP Lox^+/C285F^ (Figure 3C).

To look for a sex hormone effect, we castrated male HBP Lox^+/C285F^ mice. We found lower SBP in the castrated male HBP Lox^+/C285F^ mutants, relative to the sham HBP Lox^+/C285F^ mice (Figure 3D, *p* < 0.05), and similar to HBP Lox^+/C285F^ females. Aortic caliber followed a similar trend (Figure 3E).

### 2.3. Increased Number of Breaks and Irregular Elastic Lamellar Sheets in Lox^+/C285F^ Mice

**Figure 4:**
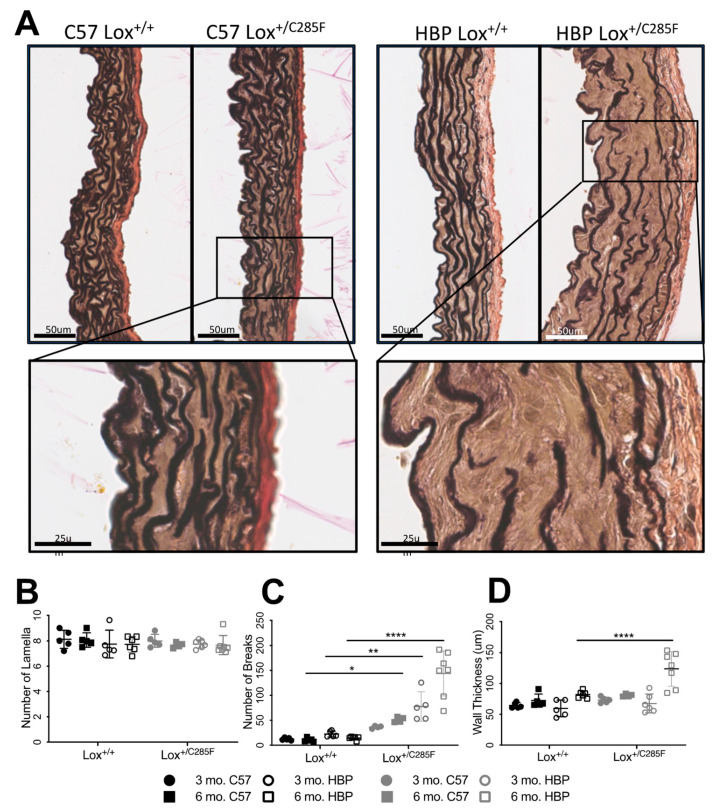
Increased elastic lamellar breaks in Lox^+/C285F^ mice. (**A**), EVG staining of 6-month C57, and HBP Lox^+/C285^ aortas revealed largely intact but disorganized elastic lamellae. Enlarged insert in (**A**) demonstrates the presence of breaks in Lox^+/C285F^. (**B**–**D**), Quantification of the number of lamella (**B**), breaks (**C**), and wall thickness (**D**) in Lox^+/+^ and Lox^+/C285F^ mice under both C57 and HBP backgrounds showed an increasingly disorganized and thick vascular wall, most obvious in the 6-month HBP Lox^+/C285F^ (Lox effect *p* < 0.0001, age effect *p* < 0.05 and HBP effect <0.001, multivariable regression, see Supplemental Table S1. * *p* < 0.05; ** *p* < 0.01; **** *p* < 0.0001.

Looking at the mutant aortas histologically using EVG, which stains elastin black, we noted a somewhat disorganized elastic lamellar structure in the (male) C57 Lox^+/C285F^ mice (Figure 4A). No difference in lamellar number was noted, regardless of the Lox genotype, genetic background, or age (Figure 4B). However, breaks were present in all elastic lamellae, including the internal elastic lamina (IEL). By two-way ANOVA and subsequent Sidak’s multiple comparison testing, the Lox^+/C285F^ effect on breaks is not statistically significant in the C57 background at 3 months (Figure 4C) but is more obvious in the dilated vessels: C57 Lox^+/C285F^ at 6 months (*p* < 0.05) and HBP background at both 3 months (*p* < 0.01) and 6 months (*p* < 0.0001). While an increase in breaks is seen as early as 3 months, increased medial thickness is not noted until 6 months in the HBP Lox^+/C285F^ (Figure 4D, *p* < 0.0001). Multivariable linear regression shows that breaks are most strongly influenced by Lox genotype (*p* = 2.8 × 10^−8^) with more minor effects from age (*p* < 0.05) and genetic background (*p* = 6.8 × 10^−4^, See Supplemental Table S1). Age had the strongest effect on wall thickness (*p* = 2.5 × 10^−5^), followed by Lox genotype (*p* = 1.5 × 10^−3^). For both phenotypes, a synergistic effect was seen among Lox^+/C285F^ status, HBP background, and older age (Supplemental Table S1).

**Figure 5:**
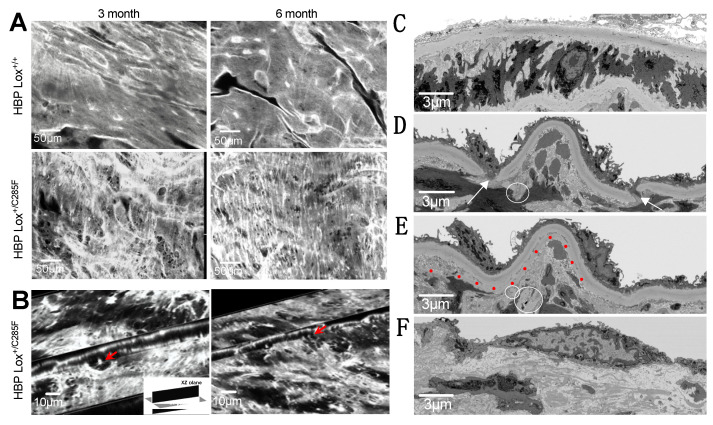
Increased fenestrations in Lox^+/C285F^ elastic lamellae. (**A**), En-face two-photon imaging shows smooth and regular IEL in Lox^+/+^ (top) while Lox^+/C285F^ are irregular and less tightly woven with numerous obvious fenestrations (bottom) See Supplemental Figure S1 for images of the second lamella). Representative images, *n* = 3. (**B**), Still images demonstrate fenestrae in 3D by crossing XY (10 μm) and XZ (5 μm) planes of the images in Lox^+/C285F^ mice (see also Supplemental Videos S1–S6). (**C**–**F**), Still images from FIB-SEM show an intact IEL in Lox^+/+^ (**C**). In contrast, Lox^+/C285F^ mice exhibit pathology ranging from significant disruptions/holes (arrows), disconnected “floating” segments (circled), and even sheared (red dotted line) stretches (**D**,**E**) to a severe, completely disorganized IEL (**F**) (See 3D reconstructed movies in Supplemental Videos S7–S9).

To further characterize the eroding aortic wall, fresh ascending aorta of 3- and 6-month HBP Lox^+/+^ and Lox^+/C285F^ were imaged using two-photon en-face imaging. The image stacks were reconstructed to produce a volumetric representation of the internal elastic lamina and the subsequent 2–3 lamellae. HBP Lox^+/+^ elastic lamina (Figure 5A) are smooth, nearly continuous elastic sheets with only occasional small fenestrae. However, like the light microscopy imaging, HBP Lox^+/C285F^ mice reveal increased fenestrations (or breaks as seen in 2D) (Figure 5A). Cross-sectional images of the XY and XZ planes confirm that these fenestrations do not merely represent an invagination of the elastic layer (Figure 5B and Supplemental Videos S1–S6). The fenestrae become more numerous with increasing age. In addition, even at 3 months, the lamellar structure in the Lox^+/C285F^ is noticeably more disorganized, with a frayed appearance that becomes more obvious with age. Similar findings are seen deeper in the vessel wall as well (Supplemental Figure S1).

When viewed at the ultrastructural level using FIB-SEM, 6-month-old HBP Lox^+/+^ mice have a smooth and continuous internal elastic lamina (IEL, Figure 5C, and Supplemental Video S7), while the HBP Lox^+/C285F^ IEL shows more discontinuity (Figure 5D,E, and Supplemental Video S8) with frequent partial thickness invaginations, fenestrations, and even disassociated “floating” elastin segments. The video reconstructions show 0 vs. 7 fenestrations in the IEL alone in a similar cross-sectional space for HBP Lox^+/+^ and Lox^+/C285F^, respectively. In some regions, the HBP Lox^+/C285F^ IEL is relatively intact, while in others, it lacks almost any organizational structure (Figure 5F and Supplemental Video S9). Overall, the Lox^+/C285F^ lamellae appear to be less tightly woven, with some fibers appearing fractured or split internally (Figure 5E and seen more clearly in Supplemental Video S8). The cells in the HBP Lox^+/+^ aortas are cuboidal and closely packed, while those from the HBP Lox^+/C285F^ are irregular with additional surrounding accumulated nonfibrillar extracellular material. In some HBP Lox^+/C285F^ cases, multiple flattened SMCs are seen layered on one another without an intervening elastic lamella.

**Figure 6:**
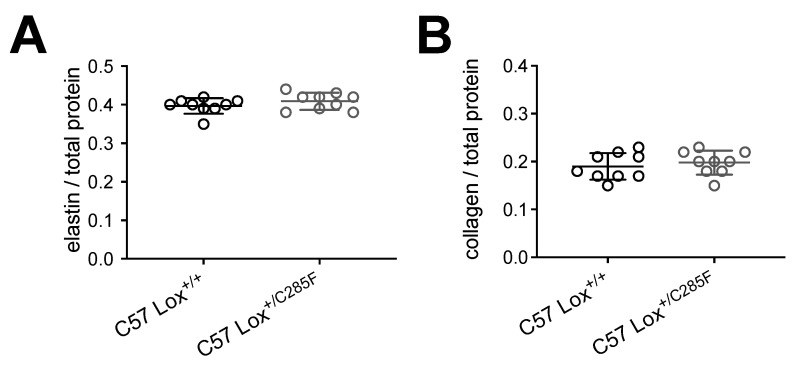
Elastin and collagen content in Lox^+/C285F^ mice. (**A**,**B**), No differences were noted in the total quantity of elastin ((**A**), *t*-test) and collagen ((**B**), *t*-test) deposited in C57 Lox^+/C285F^ vs. Lox^+/+^ aortas.

Collagen detection using fluorescence microscopy shows no obvious increase in collagen fibers (data not shown). Likewise, no difference in total insoluble elastin or collagen content was detectable by amino acid analysis (AAA) (Figure 6A,B) of hydrolyzed tissue.

### 2.4. Mechanism of Lox-Mediated Disease

**Figure 7:**
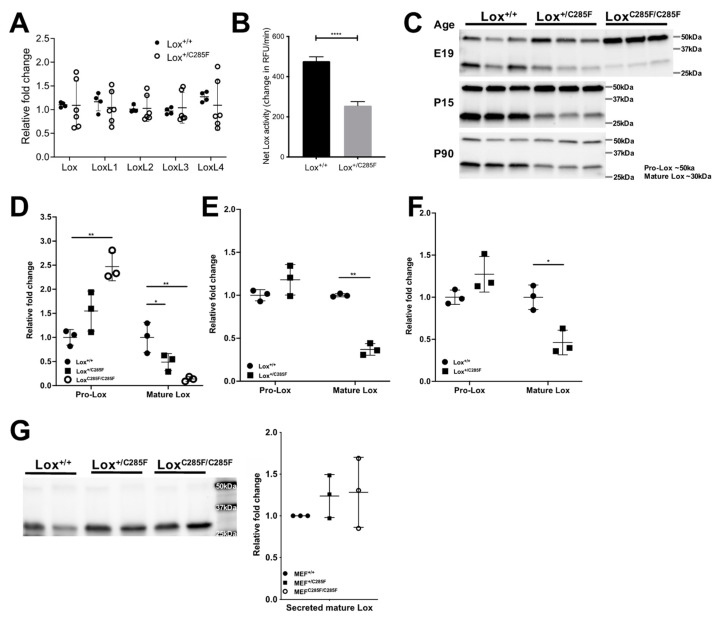
Mutation of Lox at C285F did not cause reduction in gene expression and Lox protein secretion as well as enzyme activity. (**A**), Lox and LoxL family members had similar mRNA expression in both Lox^+/C285F^ and Lox^+/+^ mice as measured by quantitative PCR (*t*-test). (**B**), Lox^+/C285F^ aorta showed 46% reduction in Lox enzyme activity in Lox^+/285F^ mice as measured by Amplex Red (*t*-test; *n* = 8). **** *p* < 0.0001. (**C**), Total lysate from frozen aortas at age E19, P15, and P90 demonstrated higher quantities of 50 kDa proLox in Lox^C285F/C285F^ as compared to Lox^+/+^ at age E19 only. However, a decreased amount of 30 kDa mature Lox in Lox^C285F/C285F^ and/or Lox^+/C285F^ was found in all three age groups. Graphs show the quantification of proLox and mature Lox at age (**D**), E19, (**E**), P15 and (**F**), P90 across all samples using total protein as normalization ((**D**) one-way ANOVA (*p* < 0.01 in both comparisons), Dunnet comparisons shown in figure; (**E**,**F**) Paired *t*-test). * *p* < 0.05; ** *p* < 0.01. (**G**) In conditioned medium collected from MEF isolated from Lox wild-type and mutant mice (*n* = 3), comparable amount of secreted mature Lox protein was detected. (Right panel, one-way ANOVA, Dunn).

To better understand the mechanism by which the copper-binding domain Lox^+/C285F^ mutant produces the phenotypes above, we evaluated gene expression and protein production. Quantitative PCR reveals similar amounts of Lox transcript in p14 aortas from HBP Lox^+/+^ and Lox^+/C285F^ mice (Figure 7A). Similarly, there is no appreciable compensatory upregulation of any of the other Lox-L genes (Figure 7A).

Protein lysates from aortas were then assessed for Lox activity using Amplex Red [19]. Lox enzyme activity was readily detected in the 3-month-old HBP aortas and showed linear kinetics (Supplemental Figure S2). In aortas from Lox^+/C285F^ mice, the rate of substrate oxidation was lower by 46% as compared to Lox^+/+^ (Figure 7B, *p* < 0.0001), indicating lower enzymatic activity.

To determine if the decreased Lox activity was due to lower total Lox protein in the C285F mutants, aortas were collected from Lox^+/+^, Lox^+/C285F^, and/or Lox^C285F/C285F^ animals at the age of E19, P15 (Lox^+/+^ and Lox^+/C285F^ only), and P90 (Lox^+/+^ and Lox^+/C285F^ only). Protein lysates prepared from these samples were used for the characterization of Lox synthesis and secretion. Lox protein is synthesized in the form of inactive zymogens. Its activation requires a functional secretory pathway and the proteolytic removal of the N-terminal propeptide to generate the 30 kDa active enzyme. Comparing genotypes by Western blot (Figure 7C), at age E19, the Lox^C285F/C285F^ aortas exhibit 14% of the average Lox^+/+^ quantity of mature Lox (Figure 7D, *p* < 0.01 in Lox^+/+^ vs. Lox^C285F/C285F^). However, there is a 2.5-fold increase in proLox protein detectable in Lox^C285F/C285F^ aortic tissue (Figure 7D, *p* < 0.01 in Lox^C285F/C285F^ vs. Lox^+/+^). For the Lox^+/C285F^ aortas at all three ages, mature Lox remains lower than in the Lox^+/+^ vessels (Figure 7D, *p* < 0.05 at E19; Figure 7E, *p* < 0.01 at P15; and Figure 7F, *p* < 0.05 at P60). There is no statistically significant difference in proLox, although an upward trend is noted in the three comparisons.

In aortic tissue, it is not possible to distinguish intra- vs. extracellular protein. To examine whether the mutant Lox protein is adequately secreted, conditioned media were collected from Lox^+/^^+^, Lox^+/C285F^, and Lox^C285F/C285F^ MEF lines, and Lox protein was quantified (Figure 7G). Minimal proLox was seen in the conditioned media, and the amount of 30 kDa mature Lox was similar across genotype groups (quantification in Figure 7G), indicating equivalent secretion of the C285F-mutated Lox protein.

### 2.5. Lox^+/C285F^ Vessels Are Susceptible to Elastolytic Damage and Show Increased Elastase Production

Together with the imaging depicting looser, less organized elastic lamellae and abnormal cell morphology, the combination of normal to reduced extracellular levels of mature Lox (depending on the system) and normal elastin and collagen concentrations suggest a change in the quality, rather than quantity, of elastic fibers and predict that the elastin deposited by Lox^+/C285F^ mutants is structurally inferior, leading to abnormal cell–matrix interactions and a more rapid failure rate.

**Figure 8:**
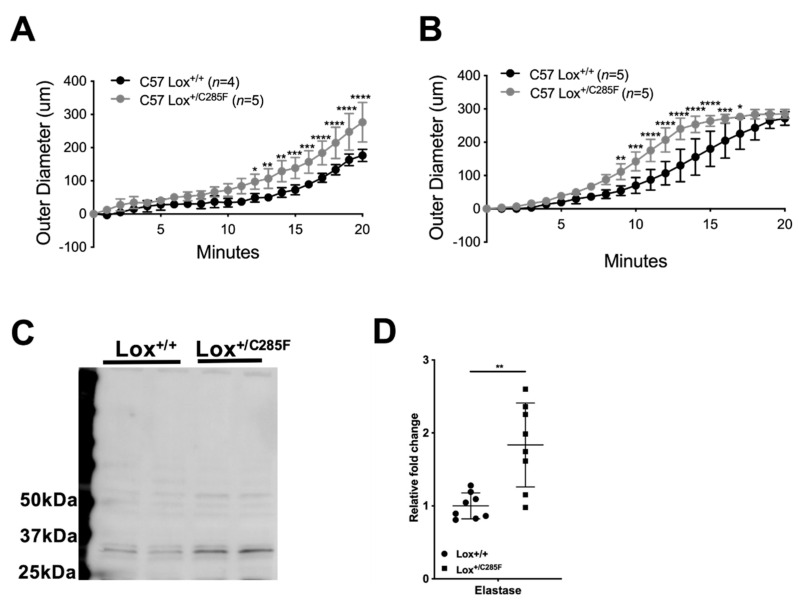
Elastin and collagen content in Lox^+/C285F^ mice. (**A,B**), When bathed in porcine elastase and mechanically stressed, the Lox^+/C285F^ ascending aorta (**A**) and left common carotid (**B**) arteries dilated at a faster rate than WT ((**A**,**B**) by two-way ANOVA, Sidak). * *p* < 0.05; ** *p* < 0.01; *** *p* < 0.001; **** *p* < 0.0001. (**C**), Increased amount of elastase was seen in the aortas of Lox^+/C285F^ mice, indicating the role of elastase in aortic dilation under Lox deficiency. (**D**), Graph shows the quantification of elastase of all measured samples using total protein as normalization. (Paired *t*-test) ** *p* < 0.01.

To test quality of the elastic matrix, we placed vessels from C57 Lox^+/+^ and Lox^+/C285F^ mice on a pressure myograph fitted with a flutter valve to allow a pulsatile flow in the vessel. We then bathed the vessel in a low concentration of elastase and monitored the vessel diameter. When assessed at pressures in the elastic portion of the curve [20,21], we found that C57 Lox^+/C285F^ mutant vessels had a more rapid increase in diameter with elastase treatment (Figure 8A (aorta; two-way ANOVA genotype effect *p* < 0.01) and B (carotid; two-way ANOVA genotype effect *p* < 0.01)).

Interestingly, we measured the endogenous elastase-1 protein level in aorta of 3-month-old HBP Lox^+/C285F^ mutants and their counterparts using freshly prepared total protein lysate. Lox^+/C285F^ aortas have more detectable Elastase-1 (Figure 8C, *n* = 8/group) (quantified in Figure 8D, *p* < 0.01 in Lox^+/C285F^ vs. Lox^+/+^).

### 2.6. Lox^+/C285F^ Gene Expression Pattern Reveals Increased Matrix Remodeling

**Figure 9:**
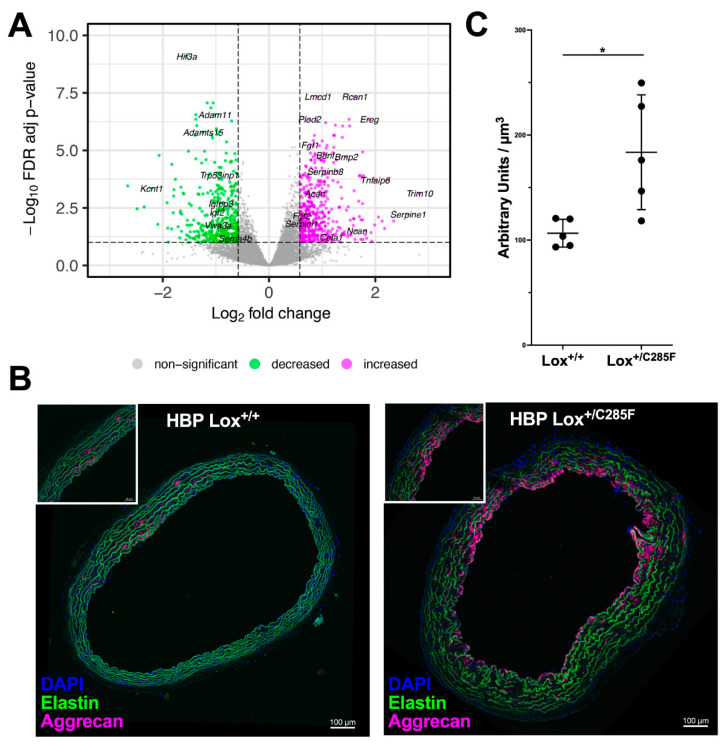
Gene expression pattern reveals increased matrix remodeling in Lox^+/C285F^ mice. (**A**), Volcano plot showing differentially expressed genes in Lox^+/+^ vs. Lox^+/C285F^ aortas (*n* = 4/group). Positive Log2 denotes higher expression in Lox^+/C285F^. (**B**), Anti−aggrecan antibody staining in 6-month HBP aortas demonstrated increasing deposition of aggrecan in Lox^+/C285F^. Representative images, *n* = 5/group. Note the asymmetric appearance of the aggrecan staining. (**C**), Quantification of aggrecan represented as total fluorescence of the intensities (arbitrary units) per each µm^3^ of aortic wall volume in Lox^+/+^ vs. Lox^+/C285F^ aortas, *n* = 5/group. * *p* < 0.05 (Paired *t*-test).

To identify additional differentially regulated pathways between Lox^+/+^ and Lox^+/C285F^ aortas, we performed RNAseq on HBP p14 aortic tissue (Figure 9A, Supplemental Table S2). Gene set enrichment showed increased expression of genes associated with the matrisome (Naba matrisome q = 4.9 × 10^−14^ and Naba matrisome associated q = 1.14 × 10^−11^) [22] along with a series of cytokine and cellular signaling transduction pathways (Supplemental Table S3) in the Lox^+/C285F^ mutants. As predicted by our protein studies, Cela1, the gene encoding elastase-1, was upregulated in the Lox^+/C285F^ aortas. In addition, several fibroblast growth factors and serpin species, as well as the proteoglycan aggrecan, were noted in the matrix and matrix-associated sublist of upregulated genes.

Aggrecan, a molecule previously noted to be increased in aneurysmal tissues [23], increases asymmetrically in the HBP 6-month Lox^+/C285F^ animals around the time we detected aortic thickening (Figure 9B,C). Looking at the genes that are decreased in Lox^+/C285F^ mutants (Supplemental Table S4), we again see enrichment for genes in the matrisome, but the significance is less robust (q = 1.70 × 10^−3^). The downregulated list includes molecules involved in insulin signaling and a set of metallopeptidases and metallopeptidase inhibitors. Interestingly, when the differentially expressed genes were analyzed using Ingenuity Pathway Analysis (IPA) to look for patterns of expression based on upstream regulators, we saw relative inhibition of genes known to be controlled by dexamethasone in Lox^+/C285F^ (z = −3.97, *p* = 9.83 × 10^−30^) as well as relative activation of genes influenced by TGFβ (z = 2.44, *p* = 7.42 × 10^−29^). See Supplemental Table S5 for a full list of regulators.

## 3. Discussion

Lysyl oxidase is a copper-binding enzyme known to cross-link elastin and collagen. The clinical literature has associated a variation in this gene with dominantly inherited FTAA (MIM#617168). In most cases of FTAA, no obvious aortic phenotype is described at birth but develops over the following years to decades. Multiple pedigrees with missense variants in close proximity to the copper-binding domain have been described, with a recently published case demonstrating an individual with a p.Cys291Ser variant, whose case was particularly severe. The mouse model used here (*Lox^+/^*^c.G854T^ (b2b370.2Clo); *Lox^+/C285F^*) impacts the analogous Cys in mice and can be used to probe disease mechanisms and to decipher the synergistic impact of influences, such as age, sex, and elevated blood pressure.

Compared to previously published Lox mutants [7,8], the vascular features in the *Lox^+/C285F^* mouse are more pronounced. Like the others, the aortas are initially of smaller caliber and display evidence of vascular stiffness (increased pulse pressure and reduced slope of the pressure–diameter curve, although the difference is noted down to 75 mmHg in the *Lox^+/C285F^* mutant and only detectable at 150 mmHg and above in the previous models). Systolic blood pressure is also increased in the *Lox^+/C285F^* relative to *Lox^+/+^* males. Over time and without pharmacologic provocation, the *Lox^+/C285F^* aorta dilates. The larger-caliber vessel is first apparent by 6 months in males and continues to progress, although frank aneurysm does not occur. This dilation may be accelerated by the introduction of a small amount of 129X1/Sv × genetic material on chromosome 1 in an otherwise C57Bl/6 genetic background. This area had been linked to a QTL for blood pressure in previous studies [24] and does raise the blood pressure of the male mice. Hypertension is a known risk factor for aneurysm progression [25], and lowering the blood pressure by treatment with either beta blockers or angiotensin receptor blockers slowed the aneurysm progression rate in patients with Marfan syndrome [26]. Likewise, as in humans [27], female *Lox^+/C285F^* mice do not develop dilation as quickly as males and are less influenced by the HBP background. Castration of male mice leads to milder phenotypes, similar to females, suggesting either a provocative role for testosterone or a protective one for estrogen in the dilation phenotype [28]. Previous studies in the *Col3A1*-related vascular Ehlers–Danlos mouse model also show sex-based differences in aneurysm [29]. In this case, both androgen and perinatal oxytocin were linked to the outcomes. We did not specifically study pregnancy as a modifier of aortic outcomes in this study, but we do note that while unmated females were used for the 3- and 6-month experiments, retired breeders were used for the 12-month studies, suggesting a possibility for similar mechanisms to play a role in the late dilation seen in the female *Lox^+/C285F^* animals.

On the molecular level, our studies showed that the mutant message is stable, and full-length protein is produced by cells. Moreover, the *Lox^+/C285F^* protein was adequately produced, secreted, and cleaved such that the conditioned media from MEFs contained normal to increased amounts of mature 30 kDa protein. *Lox^+/C285F^* aortic tissue, on the other hand, contained relatively less mature enzyme, suggesting the possibility of increased turnover of the mutant mature protein in the in vivo setting.

Previous studies have shown the importance of Lox’s Cys pairs for positioning the molecule’s CBD relative to the LTQ [6,30]. As such, even if increased turnover does not occur, the loss of a Cys proximal to the CBD can reasonably be expected to directly impact activity. Indeed, the activity assay shows a marked reduction (−46%) in Lox activity in heterozygous aortic tissue. Nevertheless, altered kinetics of enzyme activation or release from the developing elastic fiber could also be considered. To become active, Lox must be targeted to the elastic fiber by way of its prodomain [31]. Once there, its prodomain is removed by a procollagen C peptidase, activating it. It was previously reported that BMP1′s ability to process type 1 procollagen is enhanced by its binding to fibronectin [32], and fibronectin is known to interact with the C-terminal portion of Lox [33]. As such, it is possible that, in addition to influencing the catalytic function of the enzyme directly, the variation that impacts the C-terminus may also impact the complex interaction among the molecules required for either Lox’s activation or the duration of its occupancy on the elastic fiber, invoking a dominant negative mechanism.

However, how does that decreased activity produce the aneurysm phenotype? Considering our light, two-photon, and scanning electron microscopy together, we first see increased elastic lamellar fenestrations in the *Lox^+/C285F^* mutant, followed by later vessel wall thickening and loss of normal smooth muscle cell appearance. These holes are subtle in young animals and increase with age and HBP influences. Studies in the *Fbn1* ^C1039/+^Marfan mice also show increased fenestrations [34], but unlike the Marfan mice, in which the elastic lamellae remain smooth and regular by two-photon imaging, the *Lox^+/C285F^* lamellae appear increasingly ragged. The elastic sheet appears less tightly woven, with a disorganized and frayed façade, even at early ages, suggesting that although the apparent vascular defect is mild at young ages, the elastic fibers are not deposited normally. That initial defect is then amplified with increasing time and pressure that unravels the imperfectly woven elastic sheet.

In addition, our protease experiments reveal the Lox^+/C285F^ mutants to be overly susceptible to proteolytic damage, with more rapid dilation in response to elastase treatment than in *Lox^+/+^* aortas. Intriguingly, the *Lox^+/C285F^* vessels themselves exhibit increased elastase. In many aneurysm models where the ECM is obviously abnormal, the tissue attempts to compensate by remodeling. Correspondingly, our gene expression studies show enrichment for matrisome genes, as well as genes known to be regulated by TGFβ and dexamethasone. Perturbation of TGFβ [35,36] is well-known in aneurysm models, and dexamethosone [37,38] is known to influence elastic fiber assembly.

The FIB-SEM imaging takes this a step further, revealing not only fenestrations, but also disconnected and fractured sections of elastic fibers in some areas of the *Lox^+/C285F^* aorta and a complete loss of the lamellar structure in others. What is also prominent in these samples from older mice is the loss of normal smooth muscle cellular structure and the accumulation of a nonelastin ground substance in the interlamellar space. Studies have suggested that proteoglycan accumulation is associated with vessel rupture and dissection risk [23,39,40,41]. Aggrecan, in particular, was found to be accumulated in a mouse model of severe Marfan syndrome [23,42]. These mice died from thoracic aortic aneurysm and dissection (TADD) early with an average survival of 2.5 months and demonstrated increased accumulation of aggrecan when compared to their wild-type littermates. Most importantly, the mice that died of aortic rupture exhibited the highest amount of aggrecan accumulation, which spanned the full thickness of the ascending aorta. This demonstrated that the degree of aggrecan accumulation, either due to increased production or decreased degradation, correlates with the severity of aortic dilation and rupture [23]. In our immunofluorescence experiments, increased aggrecan deposition was detected in tissues of 6-month-old HBP *Lox^+/C285F^* mice, corresponding with the period of prominent aortic wall thickening and dilation in this genotype. The aggrecan in the Lox^+/C285F^ mutant is deposited asymmetrically, similar to what is seen in mice with severe Marfan syndrome [23] as well as in *Eln^+/-^* mice [43] where its location was thought to be impacted by differential mechanical forces, suggesting an attempt at a compensatory response to the elastic fiber destruction [23].

**Figure 10:**
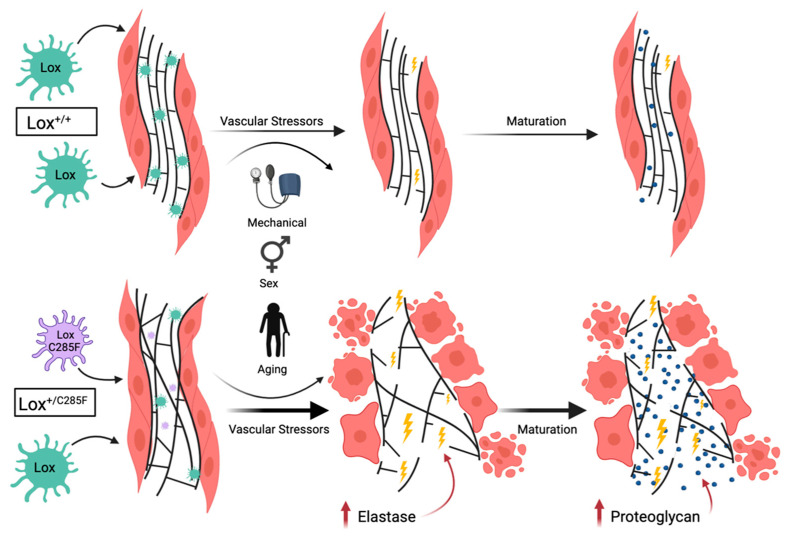
Lox C285F model results in structurally incompetent elastic fibers that are more susceptible to proteolytic damage causing progressive elastic lamellar damage and altered cell–matrix interactions. In the top row, *Lox^+/+^* mice exhibit structurally competent elastic lamella (green Lox enzyme) with the expected mild degradation that accompanies aging and a lifetime of the mechanical stress associated with repetitive stretch–recoil cycles. The *Lox^+/C285F^* mutant mice, however, deposit structurally incompetent elastic fibers due to the mutated form of the enzyme (smaller purple Lox C285F with reduced enzyme activity). The abnormal lamellae are unusually susceptible to proteolytic damage and undergo increased destruction that is amplified by elevated blood pressure, male sex, and aging. An uptick of elastase (yellow lightning bolt), a morphologic change in the smooth muscle cells, and infiltration of the elastic lamellae with proteoglycans (blue dots) occur as part of the process. Of note, the aggrecan deposition occurs somewhat later, after elastic fiber breaks are more numerous. Although the deposition of proteoglycans may initially be a compensatory response, the molecules may ultimately further disrupt cell–matrix interactions leading to further vascular wall dysfunction.

Taken together, these studies extend the body of data linking missense Lox variants proximal to the copper-binding domain with aortic disease. The data support a mechanism (Figure 10) whereby the *Lox^+/C285F^* mutants deposit structurally incompetent elastic fibers that are more susceptible to degradation with time and environmental stressors. Increases in elastase and aggrecan accumulation in the extracellular matrix further disrupt the incompetent elastic fibers and cause aortic dilation, resulting in aortic diseases. As such, patients with pathogenic variation within this domain are at increased risk of aortic disease that increases with age and may benefit from disease-modifying therapies, such as blood pressure control [27], with consideration for potential hormonal strategies requiring further investigation.

Our study demonstrated a potential mechanism by which Lox variants near the copper-binding domain induce changes to the aortic architecture and function. The process is impacted by complex interactions between elastic fibers and smooth muscle cells over time and in response to ongoing physiological stress. To truly understand this process, it would be informative to perform multimodal physiological and imaging analyses in the same animals over time; such work may illustrate important associations between real-time physiological parameters on aortic outcomes. Of particular interest in understanding the sex effects would be an investigation of pregnancy-associated outcomes in females. On the molecular side, previous investigators have noted changes in multiple Lox subtypes in the walls of aneurysmal vessels [44]; our study does not show changes in mRNA expression of the various Lox types in this mutant, but protein level quantification would be needed to rule this out. Likewise, proteomic investigation of the vessel wall may provide further insight into the impact of proteolytic damage to the vessel wall. Information about the quantity and quality of elastin crosslinks remains unknown.

In conclusion, missense variants near the copper-binding domain produce highly penetrant, early-onset autosomal dominant thoracic aortic aneurysm in humans. Work presented here shows that mutations of this type in mice produce a secreted protein that generates irregular elastic fibers that are increasingly susceptible to proteolytic damage over the lifetime of the animal, leading to aortic dilation. Complex physiological factors, such as sex, blood pressure, and age, influence these effects. Additional work is needed at the population level to ascertain the impact of variants further away from the copper-binding domain.

## 4. Materials and Methods

### 4.1. Mouse Strains and Breeding

Experiments were approved by the NHLBI animal studies committee. The *Lox^+/^*^c.G854T^ mouse (MGI 5313524(b2b370.2Clo); JAX #013616- C57Bl/6J-b2b370Clo; Lox*^+/C285F^*) was created through the Bench to Bassinet program using ENU mutagenesis of C57Bl/6 mice [15]. Two mutations were present in the parental line (b2b370.1Clo and b2b370.2Clo, http://www.informatics.jax.org/allele/MGI:5313524, accessed on 22 April 2022). The Lox variant (b2b370.2), when present in homozygous state, causes stenosis of the great arteries, cardiac hypertrophy, and diaphragmatic hernia. The identity of b2b370.1Clo was never identified genetically; mice homozygous for the b2b370.1Clo allele exhibited holoprosencephaly, right-sided aortic arch, and hypoplastic proximal arteries. Sperm from males that had produced *Lox^G854T/G854T^* offspring were stored at Jackson labs for further study. It is unknown whether these fathers also carried the unknown b2b370.1Clo allele. The line studied here was rederived at Jax by fertilization of C57Bl/6J eggs with these sperm. The resultant pups were genotyped using TaqMan SNP Genotyping Assay kit (ThermoFisher Scientific, Waltham, MA, USA, Assay number 4332077 custom: mLoxG854T) and were backcrossed further to C57Bl6/J to decrease the likelihood of cotransmission of the second unknown allele. After more than 10 generations of backcrossing or sibling breeding, no mice with holoprosencephaly were identified. Consequently, the line reported here is thought to represent only the effect of *Lox* variation. We refer to this mouse throughout the paper as C57 *Lox^+/C285F^*.

To increase blood pressure, the C57 Lox*^+/C285F^*mouse was bred to a majority C57Bl/6 mouse strain that also carries 129X1/Sv material in a specific region of chromosome 1 (minimal interval for 129/Sv material Chr1: 086182722-137503552 and maximum interval Chr1:082250512-140519860 (NCBI37/mm9 numbering)) based on SNP genotyping in the region. Mice with 129X1/Sv genetic material in this region were shown in quantitative trait locus studies to have higher blood pressure, with homozygosity for 129X1/Sv raising blood pressure by 15–20 mm Hg over C57Bl/6J [24]. Mice carrying homozygous 129X1/Sv material on chromosome 1 in this area are referred to as HBP *Lox^+/+^* or HBP *Lox^+/C285F^*.

For the described experiments, mice were phenotyped at three, six, or twelve months of age. Unmated littermates were used whenever possible, although not all animals could be assessed for all phenotypes. Animals were housed in group cages under standard conditions.

### 4.2. Systemic Blood Pressure and Heart Rate Measurement

Blood pressure was measured as previously described [45]. Briefly, once a level plane of anesthesia was achieved with isoflurane (Isoflurane florane, Baxter, Deerfield, IL, USA), a pressure catheter (1.0-F, model SPR-1000, Millar Instruments, Houston, TX, USA) was inserted into the right carotid and advanced to the ascending aorta. Systolic and diastolic pressures were recorded using Chart 5 software (ADInstruments, Sydney, Australia). Animals were monitored closely for discomfort or over-sedation.

### 4.3. Castration

At postnatal day 20, mice were anesthetized with 1–3% isoflurane. Hair was removed from the abdomen. The skin was prepared with surgical scrub/alcohol, and aseptically draped. A midline skin incision was made on the lower ventral abdomen to expose the abdominal cavity. Each vas deferens was then identified to locate the testicles. Testicles were moved into the abdominal cavity and dissected free from the interior scrotal wall. The artery and vein were cauterized proximal to the testicles, and the testicles were removed. The abdomen was closed using a simple uninterrupted suture and the skin closed with clips. Mice were given 1mg/kg Buprenorphine subcutaneously pre-operatively (ZooPharm SR-LAB, Fort Collins, CO, USA) and 4–5 drops of Bupivacaine (Fresenius Kabi, 460417, Lake Zurich, IL, USA) on the wound prior to closing the skin and allowed to recover. All animals were monitored post-op for pain or discomfort.

### 4.4. Histology

The ascending aorta was perfused with phosphate buffered saline (PBS), excised, and fixed in 1 mL of 10% buffered formalin (Fisher Scientific, SF100-4, Waltham, MA, USA) for 24 h, before dehydration in ethanol. Vessels were then embedded in paraffin and cross-sectional rings cut from just proximal to the innominate down to the root. Representative sections along this stretch were stained with Elastic Tissue Fibers-Verhoeff Van Gieson (EVG, Poly Scientific R&D, k059, New York, NY, USA) stain according to the manufacturer’s instructions to visualize elastin. Slides were scanned on a NanoZoomer 2.0-RS digital slide scanner (Hamamatsu Photonics, Hamamatsu City, Japan) and analyzed using NDP.view2 (Hamamatsu Photonics, Hamamatsu City, Japan) viewing software. In each quadrant, lamellar number was manually counted and wall thickness measured using a preinstalled ruler in the NDP.view2 software. Total number of breaks in the elastic lamella were also manually quantified in each section.

### 4.5. Two-Photon Microscopy Imaging and Analysis

A 120 µm thick adhesive spacer (Electron Microscopy Sciences, 70327-8S, Hatfield, PA, USA) was placed on a glass slide, and 9 µL of PBS was pipetted in the center. The ascending aorta was then dissected from each mouse and rinsed with PBS to remove blood. The vessel was immediately cut lengthwise, opened flat, and mounted with intima (endothelial cell side) facing up. A #0 cover slip (Electron Microscopy Sciences, 72198) was placed gently on top on the tissue, which allowed en-face imaging of the vessel surface. PBS was used as mounting media. With the optimized setup [46], acquisition of images consisted of an en-face z-stack of images running from intima to about 100 μm depth (or about 4 elastic lamellae) where the elastin autofluorescence signal became too low for detection.

Two-photon microscopy imaging using an inverted Leica SP5 five channel confocal and multiphoton MP-OPO system (Leica Microsystems, Mannheim, Germany) was performed as previously described [46]. Two-photon mode was used with a pulsed femtosecond Titanium:Sapphire (Ti:Sa) laser, (Chameleon Vision II, Coherent, Santa Clara, CA, USA) tunable for excitation from 680 to 1080 nm. Imaging of freshly prepared, whole mounts “en-face” aorta preparations was performed using Leica HC-PL-IRAPO 40X/1.1 NA water immersion objective (WD = 0.6 mm). Two-photon excitation at 860nm was used to reveal structural information by intrinsic contrast imaging of second harmonic generated signal (SHG) collected via 525/40 nm emission filter on nondescanned detectors 1 (NDD1). Aorta autofluorescence from elastin was collected with 525/40 nm emission filter on NDD2.

For 3D volume rendering, series of xyz images (typically 1 × 1 × 1.5 μm^3^ voxel size) were collected along the *z*-axis at 1.5 μm intervals over a range of depths (80–120 μm) throughout the depth of whole mount tissue and over large regions using the tile function of the Leica LAS-AF software to automatically generate stitched volumes comprising an area of approximately 2.0 × 1.2 mm^2^ (x-y) and 100 μm (z). For 3D renderings and quantitative image analyses, we used Imaris v 9.5.1 software (Bitplane Inc., Zurich, Switzerland).

### 4.6. Focused Ion Beam-Scanning Electron Microscopy (FIB-SEM)

For the aorta samples, *Lox^+/+^* and *Lox^+/C285F^* were processed as previously described [47], with the exception that a 30 min room temperature pyrogallol incubation (320 mM aqueous solution, pH 4.1, Alfa Aesar, Fisher Scientific, 44152-09) was used instead of thiocarbohydrazide in the staining process. The samples were imaged using a Zeiss Crossbeam 540 FIB-SEM microscope (Carl Zeiss Microscopy GmbH, Jena, Germany). Platinum and carbon pads were deposited over the region of interest (ROI), and the run was set up and controlled by Atlas software (Fibics Incorporated, Ottawa, ON, Canada). ROI were selected from *Lox^+/+^* (*n* = 1) and *Lox^+/C285F^* aortas (*n* = 2). SEM settings: 1.5 kV; 1.5 nA; milling probe: 700 pA. The slice thickness and the pixel size were set to 9 nm.

### 4.7. Advanced Imaging and Analysis

The FIB-SEM images were aligned using Atlas software. The data were then imported into Fiji software (Image J) [48] and binned 3X, to 27 × 27 × 27 nm isotropic voxels. The contrast was then normalized using the Enhance Local Contrast (CLAHE3D) [49] plugin in ImageJ [50]. Images and videos were rendered using Imaris v 9.3.1 (Bitplane Inc., Zurich, Switzerland).

### 4.8. Biochemical Analysis on Elastin and Collagen Contents

Aortas from the aortic root to the innominate were dissected from WT and mutant mice and stored at −80 °C prior to processing. Briefly, specimens (about 20 mg wet weight) were thawed and digested with high purity bacterial collagenase (Sigma, C0773; 100 U/mL, 37 °C, 18 h). After centrifugation, the soluble fractions containing collagen were hydrolyzed in 6 N HCl at 110 °C for 24 h and subjected to amino acid analysis (AAA) on a Biochrom 30 amino acid analyzer according to standard machine specific protocols (Biochrom, UK) using specified amino acid standards (Onken, Germany). The respective protein content of the different fractions was calculated as the sum of molecular weights of each amino acid (aa) in the AAA corrected for the molecular weight of water released during peptide bond formation. Collagen content was calculated based on a content of 14 mg hydroxyproline in 100 mg collagen. The insoluble fraction after collagenase digestion was extracted by hot alkali (0.1 N NaOH, 95 °C, 45 min). After centrifugation, an aliquot of the supernatant containing noncollagenous, nonelastin proteins and an aliquot of the insoluble residue containing insoluble elastin were subjected to hydrolysis and AAA as outlined above. For analysis, both collagen and elastin were normed to total protein, which was calculated as the sum of elastin, collagen, and noncollagenous, nonelastin proteins in the aorta.

### 4.9. RNA Preparation for QPCR and RNA Seq

*Lox^+/+^* or *Lox^+/C285F^* mouse aortas were isolated by dissection from 2-week-old males and flash frozen in liquid nitrogen and stored at −80 °C. RNA was isolated using a Qiagen RNA Extraction kit (RNeasy, Qiagen 74104, Hilden, Germany). Briefly, frozen tissues were thawed in a supplied lysis buffer also containing β-mercaptoethanol. Tissues were disrupted in a bead homogenizer (Bead Ruptor 4, Omni International, 25-010, with 2 mL bead tubes Omni International, 19-628) and then processed according to the manufacturer’s protocol. RNAs were validated for quantity by nanodrop and for integrity by bioanalyzer. For quantitative PCR (qPCR) analysis, cDNAs were generated from RNA by reverse transcription according to the manufacturer’s protocol (High-Capacity cDNA Reverse Transcription kit ThermoFisher Scientific, 4368814). Three *Lox^+/+^* and three *Lox^+/C285F^* cDNA samples were assayed by qPCR for Lox, Loxl1, Loxl2, Loxl3, and Loxl4 (see Supplemental Table S6 for primer sequences). qPCR assays were performed on a QuantStudio3 real-time PCR system (ThermoFisher Scientific) and analyzed by ΔΔCt with Hprt and 18S RNA as endogenous controls. For RNA seq, sequencing libraries were constructed from 300 ng of total RNA using the TruSeq Stranded Total RNA kit with Ribo-Zero Globin (Illumina 20020612, San Diego, CA, USA) following the manufacturer’s instructions. The fragment size of RNAseq libraries was verified using the Agilent 2100 Bioanalyzer (Agilent G2939BA, Santa Clara, CA, USA), and the concentrations were determined using Qubit fluorometer (Life Technologies Q33216, Carlsbad, CA, USA). The libraries were loaded onto Illumina HiSeq 3000 for 2 × 75 bp paired-end read sequencing. Fastq files were generated using the bcl2fastq software (Illumina) for further analysis. Sequence reads were aligned to mouse reference genome M16 by STAR [51], indexed using samtools, and counts were performed using the featureCounts utility of the Subread package [52], and raw counts were normalized and analyzed for differential expression using DESeq2 [53]. Genes were selected as differentially expressed with |log_2_(fold change)| > 0.58 (1.5 fold change) and false discovery rate < 0.10. Gene Set Enrichment Analysis (GSEA) of that set for genes in canonical pathways was performed using the GSEA tool and molecular signatures database v7.1 [54,55]. A total of 859 differentially expressed genes are mapped as gene symbols to 600 genes in the GSEA database. Using GSEA, *p* values were computed using the hypergeometric distribution (Fisher’s exact test) and adjusted with the Benjamini and Hochberg [56] method. Upstream regulator prediction from differentially expressed genes was performed using IPA (Qiagen Inc., https://www.qiagenbioinformatics.com/products/ingenuity-pathway-analysis, accessed on 3 January 2020). Expression data were mapped into the IPA database using Ensembl IDs, matching 857 of 859 differentially expressed genes.

### 4.10. Lysyl Oxidase Enzyme Activity Assay

The aorta, from the root to diaphragm, was dissected out and snap frozen in liquid nitrogen. Lox enzyme activity was measured in tissues as described by Trackman and Bais [19] by measuring the production of hydrogen peroxidase through oxidation of Amplex Red (ThermoFisher Scientific, A12222, Waltham, MA, USA), which results in the generation of highly fluorescent resorufin for detection. Briefly, the frozen aortas were homogenized using the Bead Ruptor 4 Homogenizer (Omni International) in 250 µL of buffer containing 6M urea and 50 mM borate (pH 8.2). One hundred microliters of samples were then added to the reaction buffer with final concentration of 1.2M urea, 50 mM borate (pH 8.2), 1 unit/mL of horseradish peroxidase, 12.5 µM Amplex Red, and 12.5 mM 1,5-diaminopentane. Each sample was tested in duplicate. Parallel assays were prepared with 625 mM β-aminopropionitrile (BAPN) to inhibit the Lox activity. All reactions were incubated at 37 °C and measured for fluorescence at excitation wavelength of 563 nm and emission wavelength at 587 nm continuously for 60 min in CLARIOstar microplate reader (BMG Labtech). Reported activity is the average slope of the line generated from plotting the resorufin fluorescence by time done in *n* = 8.

### 4.11. Isolation and Protein Extraction from Mouse Aorta for Western Blot

Mice were sacrificed by CO_2_ inhalation, and the vasculature was perfused with phosphate-buffered saline, via the left ventricle, to remove all blood. The aortas from E19, P15, and P90 were then carefully excised (free of fat) from the root down to the diaphragm. Vessels were snap frozen in liquid nitrogen and stored at −80 °C. For Western blot analysis, the frozen aortas were then homogenized using the Bead Ruptor 4 Homogenizer (Omni International) in 200 µL of RIPA buffer (Milliporesigma, R0278, Burlington, MA, USA) containing complete EDTA-free Protease Inhibitor Cocktail (MilliporeSigma, 11873580001, Burlington, MA, USA), and protein was quantified with Pierce Rapid Gold BCA Protein Assay kit (Thermo Scientific, A53226). A total of 30 µg of total protein was loaded on TGX stain-free protein gels (Bio-Rad, 17000927, Hercules, CA, USA) for blotting.

### 4.12. Isolation of Mouse Embryonic Fibroblasts (MEFs)

A pregnant *Lox^+/C285F^* mouse was sacrificed at 13 days postcoitum. The uterus was then dissected out and rinsed in 70% ethanol and placed in sterile PBS. Each embryo was then separated away from the uterus under sterile conditions and decapitated. The head and internal organs were removed, and the remaining carcass was washed in PBS and finely minced using a sterile razor until segments were small enough to pipette. The minced fragments were then digested with 0.05% trypsin/EDTA (ThermoFisher Scientific, 25300054, Waltham, MA, USA) and 100 Kunitz units of DNase I (ThermoFisher Scientific 18047019, Waltham, MA, USA) at 37 °C for 15 min. After that, trypsin was inactivated with MEF medium, containing 10% fetal bovine serum (FBS) (GE Healthcare, SH30071.03HI, Chicago, IL, USA), 1% of Penicillin-streptomycin (ThermoFisher Scientific, 15070063, Waltham, MA, USA) and 1% of nonessential amino acid (ThermoFisher Scientific, 11140050) in DMEM with 4.5 g/L glucose. The slurry was centrifuged at low speed (300× *g*) for 5 min. Supernatant was discarded, and the cell pellet was resuspended in MEF medium and seeded in one T75 flask coated with 0.1% gelatin (Stemcell technologies, #07903, Vancouver, BC, Canada). Cells reached 80–90% confluent after 48 h and were split in 1:4 ratio to obtain P1 MEFs. P1 MEFs were collected for DNA extraction to confirm the genotype and were frozen for future experiments.

### 4.13. Protein Extraction from MEFs

Passage 3 MEFs were seeded and maintained in 100 mm culture dish as above until confluent. Twenty-four hours before collection, culture medium was replaced with DMEM without phenol red and serum and was supplemented with 50 µg/mL ascorbic acid. Conditioned medium was collected after 24 h and concentrated 40-fold using Amicon Ultra-15 Centrifugal Filter Units (MilliporeSigma, UFC901024, Burlington, MA, USA). Buffer exchange with buffer containing 25 mM Tris-HCL pH8.0 and 25 mM NaCl was achieved using Amicon Ultra-0.5 mL centrifugal filter units (MilliporeSigma, UFC501008). Concentrated and buffer-exchanged medium were loaded into TGX stain-free protein gels (Bio-Rad, 17000927, Hercules, CA, USA) for blotting. The remaining attached cells were lysed with Pierce IP Lysis Buffer (ThermoFisher Scientific, 87788) containing complete EDTA-free Protease Inhibitor Cocktail (MilliporeSigma, 11873580001), and protein was quantified with Pierce Rapid Gold BCA Protein Assay kit (ThermoFisher Scientific, A53226).

### 4.14. Western Blotting

Concentrated medium or tissue lysates were added to 4× Laemmli protein sample buffer (Bio-Rad, 1610747, Hercules, CA, USA) to a final concentration of 1×. Mixtures were then incubated at 100 °C for 5 min. Denatured protein was resolved in 4–15% Mini-Protean TGX stain-free gels (Bio-Rad, 17000927) and immunoblotted on low fluorescence PVDF membrane (Bio-Rad, 1704274). Protein was detected using primary antibodies against Lox at c-terminal end (1:1000) (Abcam, ab174316) or Elastase-1 (1:1000) (Abcam, ab231117, Cambridge, UK). IRDye 680RD donkey anti-rabbit secondary antibody (1:5000) (Li-Cor, 926-68073) was used for detection. Fluorescence images were collected using ChemiDoc MP Imaging System (Bio-Rad). Quantifications on band intensity normalized to total protein loaded were done using Image Lab software (Bio-Rad) [57,58,59].

### 4.15. Pressure–Diameter Testing with and without Elastase Treatment

Similar to previous studies [45], ascending aortas were removed from *Lox^+/+^* and *Lox^+/C285F^* mice, mounted on a pressure myograph (Danish Myotechnology, Copenhagen, Denmark), pressurized, and longitudinally stretched three times to in vivo length [60] prior to data capture. Intravascular pressure was then increased in 25 mmHg steps (from 0 to 175 mmHg), and outer diameter was assessed. To calculate the change in diameter with time, the average diameter of the 3-month C57 *Lox^+/+^* or *Lox^+/C285F^* aortas at 100 mmHg was subtracted from the relevant 6- or 12-month average 100 mmHg measurement to allow quantification of the difference. A similar comparison was performed between the 6-and 12-month means. Because the same animal was not sampled at each interval, pairwise comparisons could not be performed, limiting the ability to statistically test this difference. As such, only a difference of means was presented. For the elastase treatment, a second cohort of vessels was mounted on the myograph and then cyclically inflated from 25 mmHg to 120 mmHg at 40 cycles/min utilizing the built-in flutter valve. The duty rate was set to 70/30 such that a square sine wave could reliably be achieved at the lower pressure. Upon steady state, elastase (Worthington Biochemical Corporation, LS006365 Lot:2017, Lakewood, NJ, USA) was added to the myography chamber achieving a final activity of 0.5 U/mL for aorta and 0.1 U/mL for carotid. Outer diameter was continuously recorded for 20 min and reported in the elastic portion of the curve [21].

### 4.16. Immunofluorescence

Ascending aortas from mice were fixed in 10% buffered formalin (Fisher Scientific, SF100-4) for 24 h, dehydrated in ethanol (PHARMCO, 111000200), cleared with xylene (Fisher Scientific, X5-500), and embedded in paraffin. Tissue slices with 5 μm thickness were then obtained. To perform immunofluorescence, slides were rehydrated with 100%, 90%, and 70% ethanol sequentially, and antigen retrieval was done with citrate buffer (pH 6) (Sigma-Aldrich, C9999) for 5 min. They were then blocked with 10% donkey serum (Jackson ImmunoResearch, 017-000-001, West Grove, PA, USA) in 0.5%BSA/PBS buffer for 1 h. The primary antibody against Aggrecan (Sigma-Aldrich, AB1031) was incubated at 1:200 dilution on the section overnight at 4 °C. Following three 10 min washes with PBS containing 0.05% Tween20, donkey anti-rabbit Alexa Fluor Plus 555 (Invitrogen, #A32754) at 1:100 dilution was added and incubated for 1 h. Coverslips were mounted using ProLong Diamond Antifade Mountant with DAPI (Invitrogen, #P36966). Images were acquired using a confocal microscope (Carl Zeiss LSM880, Jena, Germany). Quantification of aggrecan immunofluorescence was performed using Imaris 9.9.1 software (Bitplane Inc., Zurich, Switzerland) as follows: total fluorescence (sum) of the intensities (arbitrary units) of the aggrecan above background levels was determined in 3D (volume data set). The aortic wall volume (µm^3^) was calculated by segmenting a “3D surface” object based on DAPI channel. Total aggrecan fluorescence per wall volume (*n* = 5/group) was compared between aorta sections obtained from *Lox^+/+^* and *Lox^+/C285F^* mice.

### 4.17. Statistical Analysis

Statistical analyses were performed with GraphPad Prism version 8.0 for Mac (GraphPad Software, San Diego, CA, USA). Results are expressed as mean ± standard deviation (SD) or median if nonparametric testing is performed. The differences between groups were analyzed using ANOVA, student *t*-test, or Mann–Whitney test. Multiplicity-adjusted *p* values less than 0.05 following appropriate multiple correction was considered as statistically significant. Primary test and multiple testing type are as noted in the figure legends. When possible, the value for each data point is plotted. As such, the “*n*” in each experiment is discoverable based on the number of plotted points on the graph. When the graphing parameters do not permit plotting of individual points, the *n* for each experimental group is noted in the figure panels. For the histological analyses, a multivariable linear regression model was used to examine the impact of 3 independent variables Lox genotype, age (3 months vs. 6 months) and genetic background (C57 vs. HBP)) on each of the outcome variables. Outcomes (lamellar number, breaks, and wall thickness) were analyzed separately. These analyses were performed using SAS 9.4 (SAS Institute Inc., Cary, NC, USA) with a significance level of 0.05. A Bonferroni-corrected threshold alpha = 0.005 was specially applied for multivariable linear regression model to account for multiple comparison.

## Data Availability

The data presented in this study are available in Supplemental materials.

## Supplementary Materials

The following supporting information can be downloaded at: https://www.mdpi.com/article/10.3390/ijms23126749/s1.
