# Supplementary material for: Copper-Binding Domain Variation in a Novel Murine Lysyl Oxidase Model Produces Structurally Inferior Aortic Elastic Fibers Whose Failure Is Modified by Age, Sex, and Blood Pressure
Source: Int J Mol Sci. 2022 Jun 17;23(12):6749. doi: 10.3390/ijms23126749 (PMC9223555; doi:10.3390/ijms23126749)
Supplement: Supplementary file 1 [file ijms-23-06749-s001.zip › Tsang_IJMS Supplementals.pdf]

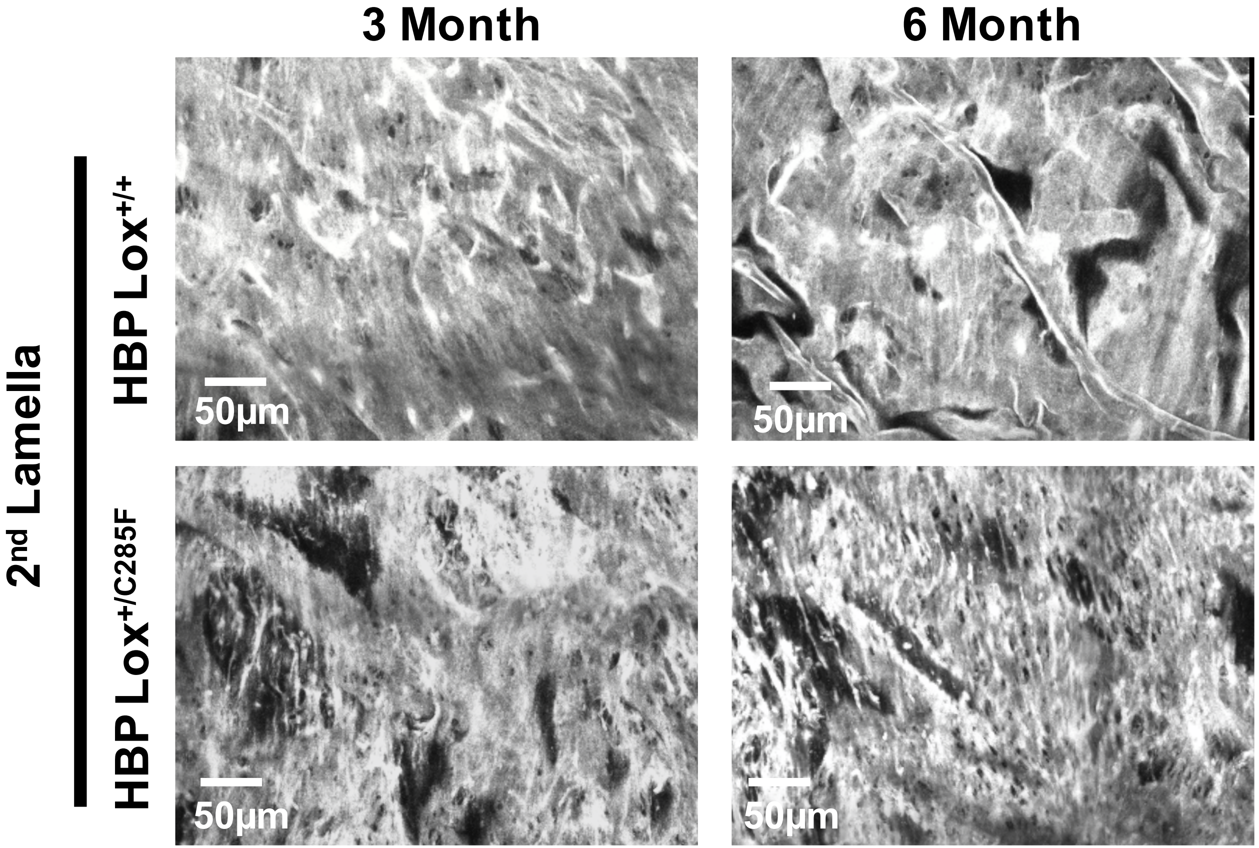

Supplemental Figure S1 Second elastic lamella in HBP *Lox*$^{+/+}$ and *Lox*$^{+/C285F}$ mice at three and six-months. En-face Two-photon imaging revealed that second elastic lamella, as in IEL (Figure 5A), have increased fenestrae density on elastic lamellar sheets in *Lox*$^{+/C285F}$ mice compared to *Lox*$^{+/+}$ at all ages. The elastic sheets in *Lox*$^{+/C285F}$ mice have a "moth-eaten" appearance not observed in *Lox*$^{+/+}$ mice. Representative images of n=3.

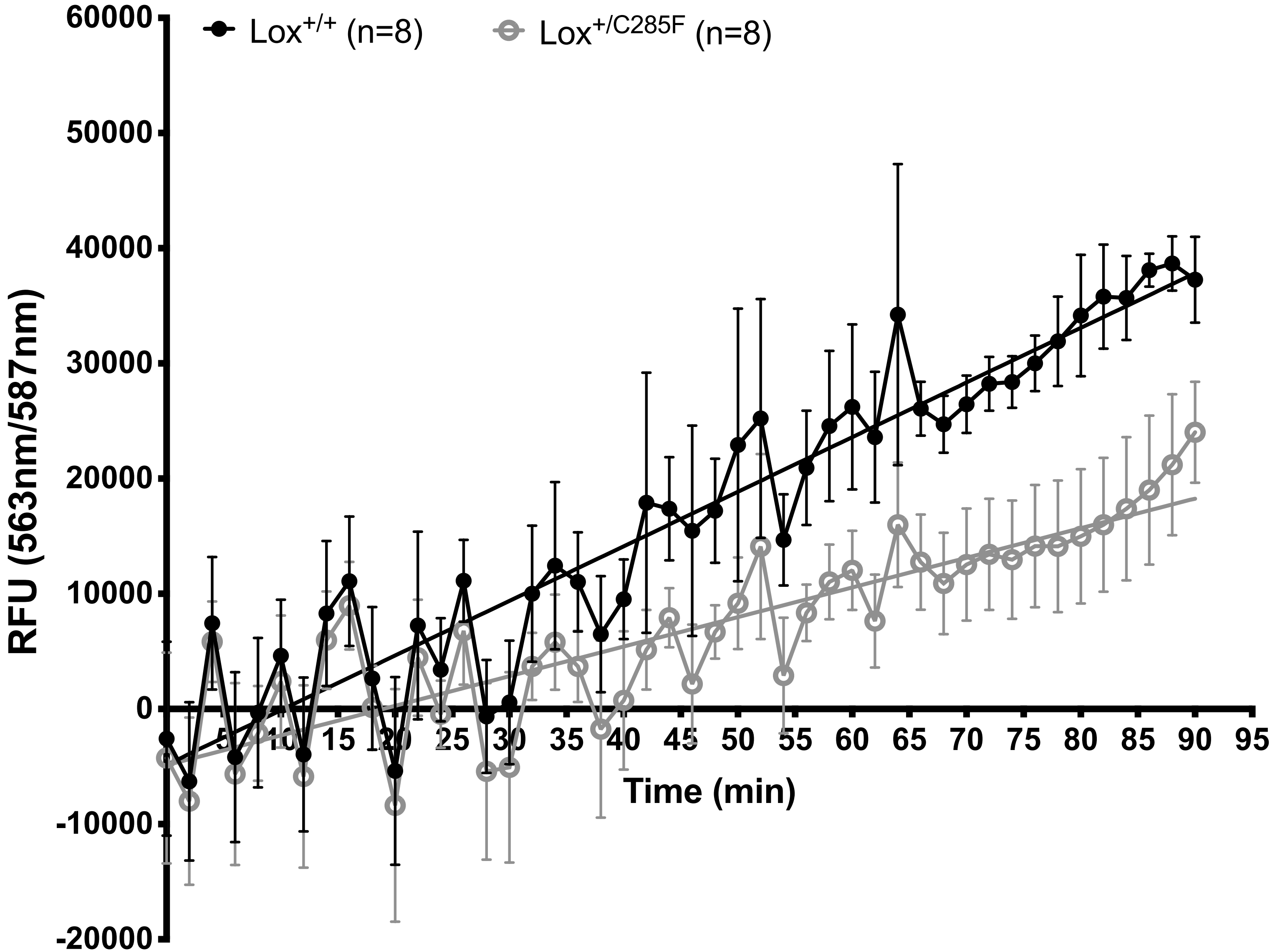

Supplemental Figure S2 Detailed kinetics of Lox enzyme activity in Amplex red assay. The assay demonstrated a decreased rate and amplitude of Lox enzyme activity in aorta from *Lox*$^{+/C285F}$ mice. The data has been normalized to background and subtracted from the duplicate reaction with BAPN (inhibitor of Lox) in replicates n=8.

**p-value**<br>Estimated group means (95%CI)	**Number of Lamella**	**Breaks**	**Wall thickness**
**Lox genotype**	**0.4926**	**2.79E-08**	**1.46E-03**
Lox $^{+/C285F}$	7.78 (7.51-8.06)	82.67 (68.25-97.09)	88.18 (80.37-95.99)
Lox $^{+/+}$	7.92 (7.64-8.19)	13.61 (-0.32-27.53)	69.36 (61.43-77.29)
**Age**	**0.5700**	**0.0497**	**2.46E-05**
3 month	7.90 (7.63-8.18)	38.12 (23.48-52.77)	65.63 (57.69-73.56)
6 month	7.79 (7.52-8.07)	58.16 (44.44-71.87)	91.92 (84.11-99.73)
**Genetic background**	**0.2082**	**6.75E-04**	**0.0638**
C57	7.97 (7.68-8.26)	29.68 (14.61-44.74)	73.49 (65.15-81.83)
HBP	7.73 (7.47-7.98)	66.60 (53.24-79.96)	84.06 (76.61-91.50)
**Group^**	**0.8727**	**6.60E-12**	**7.86E-08**
Lox $^{+/+}$ & 3mo. & C57	8.12 (7.53-8.71)	12.45 (-7.73 - 32.64)	64.48 (51.34-77.62)
Lox $^{+/+}$ & 3mo. & HBP	7.74 (7.15-8.34)	22.46 (2.28-42.64)	59.63 (46.49-72.78)
Lox $^{+/+}$ & 6mo. & C57	8.07 (7.48-8.66)	10.56 (-9.62 - 30.74)	72.38 (59.24-85.53)
Lox $^{+/+}$ & 6mo. & HBP	7.73 (7.19-8.27)	14.47 (-3.95-32.89)	82.10 (70.09-94.09)
Lox $^{+/C285F}$ & 3mo. & C57	7.99 (7.41-8.59)	36.34 (13.78-58.91)	73.26 (60.13-86.41)
Lox $^{+/C285F}$ & 3mo. & HBP	7.75 (7.21-8.29)	77.67 (57.48-97.85)	67.67 (55.67-79.66)
Lox $^{+/C285F}$ & 6mo. & C57	7.67 (7.00-8.33)	51.17 (28.61-73.74)	80.77 (66.08-95.47)
Lox $^{+/C285F}$ & 6mo. & HBP	7.66 (7.16-8.16)	144.14 (127.08-161.19)	123.68 (112.57-134.79)

^Group: the combined 8-level variable of Lox genotypes, age groups and genetic background.

Supplemental Table S1 Multivariable statistical analysis of aortic histology. Multivariable linear regression of the number of lamella, breaks, and wall thickness in *Lox* $^{+/+}$ and *Lox* $^{+/C285F}$ mice under both C57 and HBP backgrounds and different age groups. Lox genotype variables are controlled for age and genetic background, age is controlled for lox genotype and genetic background and genetic background is controlled for lox genotype and age.

This is just a placeholder - Tables uploaded individually

Supplemental Table S2 Differences between *Lox*$^{+/+}$ and *Lox*$^{+/C285F}$ gene expression. Genes selected for +/- 1.5-fold change (0.58 Log2 fold change) and FDR<10%

Supplemental Table S3 Upregulated Pathways in *Lox*$^{+/C285F}$ vs *Lox*$^{+/+}$ aortas. Gene set enrichment analysis for *Lox*$^{+/C285F}$ vs *Lox*$^{+/+}$ aortas. Below are the top 100 enriched canonical pathways in the upregulated genes (>1.5X increase, 0.1FDR), at right are the genes in each pathway with genes in each pathway indicated as "1" and non included genes as "0".

Supplemental Table S4 Down-regulated Pathways in *Lox*$^{+/C285F}$ vs *Lox*$^{+/+}$ aortas. Gene set enrichment analysis for *Lox*$^{+/C285F}$ vs *Lox*$^{+/+}$ aortas. Below are the only enriched canonical pathways in the downregulated genes (>1.5X increase, 0.1FDR) meeting at least 0.05 FDR for enrichement by GSEA (13 pathways). At right are the genes in each pathway with genes in each pathway indicated as "1" and non included genes as “0”.

Supplemental Table S5 Upstream regulators of the differentially expressed genes. Top 30 predicted upstream regulators of differentially expressed genes using Ingeunity Pathway Analysis

Gene Target	Sequence 5' to 3' Forward	Sequence 5' to 3' reverse
Lox	TGCACACACACAGGGATTGA	TGTAGCGAATGTCACAGCGT
LoxL1	TCGTCCAGTCCTGATCTGAGT	GGGTCCATGTTCACTCCCTG
LoxL2	CTTCTGCCTGGAGGACACTG	TGGGGTTAATGACAACCTGGA
LoxL3	TCTTCAGGTCGTTATCAACCCA	ACCTCCTGTTGGCTTCTTCG
LoxL4	ACAAACTGCCCCTCAGGAGT	CTGCAACATCGTTTGTGGGG
Hprt	GAGGAGTCCTGTTGATGTTGCCAG	GGCTGGCCTATAGGCTCATAGTGC
18S	GTAACCCGTTGAACCCCATT	CCATCCAATCGGTAGTAGCG

Supplemental Table S6 Primer sequences for qPCR

This is just a placeholder - Movies uploaded individually

Supplemental Video S1 Two-photon microscopy 3D reconstructed movie through a portion of 3 month old *Lox*$^{+/+}$ ascending aorta. The XY planes (10μm) move along the Z to display the appearance of elastic lamellae sheet from endothelial side towards adventitia side until about 4th elastic lamella. Representative video of n=3.

Supplemental Video S2 Two-photon microscopy 3D reconstructed movie through a portion of 3 month old *Lox*$^{+/C285F}$ ascending aorta. The XY planes (10μm) move along the Z to display the appearance of elastic lamellae sheet from endothelial side towards adventitia side until 4th elastic lamella. Representative video of n=3.

Supplemental Video S3 Two-photon microscopy 3D reconstructed movie through a portion of 6 month old *Lox*$^{+/+}$ ascending aorta. The XY planes (10μm) move along the Z to display the appearance of elastic lamellae sheet from endothelial side towards adventitia side until 4th elastic lamella. Representative video of n=3.

Supplemental Video S4 Two-photon microscopy 3D reconstructed movie through a portion of 6 month old *Lox*$^{+/C285F}$ ascending aorta. The XY planes (10μm) move along the Z to display the appearance of elastic lamellae sheet from endothelial side towards adventitia side until 4th elastic lamella. Representative video of n=3.

Supplemental Video S5 Zoom in Two-photon microscopy 3D reconstructed movie through a portion of 3 month old *Lox*$^{+/C285F}$ ascending aorta. The XY (10μm) plane move along the Z and the XZ plane move along Y to display the emergence of fenestrae. Representative video of n=3.

Supplemental Video S6 Zoom in Two-photon microscopy 3D reconstructed movie through a portion of 6month old *Lox*$^{+/C285F}$ ascending aorta. The XY (10μm) plane move along the Z and the XZ plane move along Y to display the emergence of fenestrae. Representative video of n=3.

Supplemental Video S7 FIB-SEM movie through a portion of *Lox*$^{+/+}$ ascending aorta. Movie displayed at 20FPS. Total volume acquired was 31.50 x 17.84 x 20.29 um (XYZ)

Supplemental video S8 FIB-SEM movie through a portion of *Lox*$^{+/C285F}$ (ROI1) ascending aorta. Movies displayed at 20FPS. Total volume acquired was 38.92 x 21.84 x 13.88 um (XYZ).

Supplemental video S9 FIB-SEM movie through a portion of *Lox*$^{+/C285F}$ (ROI2) ascending aorta. Movies displayed at 20FPS. Total volume acquired was 34.93 x 18.81 x 17.27 m (XYZ).
